# A case of small bowel metastasis from spinal Ewing sarcoma causing intussusception in an adult female

**DOI:** 10.1186/s12957-016-0850-4

**Published:** 2016-04-16

**Authors:** Qing Ting Tan, Jin Yao Teo, Syed Salahuddin Ahmed, Alexander Yaw Fui Chung

**Affiliations:** Department of General Surgery, Singapore General Hospital, Outram Road, Singapore, 169608 Singapore; Department of Hepatopancreaticobiliary Surgery, Singapore General Hospital, Outram Road, Singapore, 169608 Singapore; Department of Pathology, Singapore General Hospital, Outram Road, Singapore, 169608 Singapore

**Keywords:** Ewing sarcoma, Adult, Metastasis, Small bowel, Intestinal, Obstruction, Intussusception

## Abstract

**Background:**

Ewing sarcomas are highly aggressive malignant tumours occurring predominantly in the long bones of the extremities in children and young adults. About 20 % of patients will present with metastases at diagnosis with the commonest sites being the lungs, bone and bone marrow. Cases of primary small bowel Ewing sarcomas have been described but are nonetheless exceedingly rare, even more so cases of metastasis to the small bowel.

**Case Presentation:**

We describe a case of vertebral Ewing sarcoma in a 44 year-old female which metastasized to the jejunum causing intussusception.

**Conclusions:**

Ewing’s sarcoma is highly aggressive and presence of metastases, overt or subclinical, is thought to be present in almost all patients at diagnosis. As evidenced by our patient, metastatic disease can progress rapidly to cause further complications and confer a poorer survival. The possibility of metastasis, no matter how rare or unlikely the site is, should be considered and actively investigated to expedite treatment of the primary disease.

## Background

Ewing sarcomas are rare small round cell tumours that arise predominantly in children and young adults with a slight male predilection [1, 4, 7]. Ewing sarcoma most often arises in the mid-shaft or diaphysis of the long bones of the extremities with the spine making up 8 % of primary sites [2]. Approximately, 80 % of patients present with clinically localized disease although it is surmised that subclinical metastatic disease is present in almost all patients due to high relapse rates of 80 to 90 % in patients undergoing local therapy alone [3]. Metastases are mostly found in the lungs, bone and bone marrow [4]. Eight cases of primary small bowel Ewing sarcoma have been reported [8–10, 14]. One such case occurred in an 18-year-old male which was complicated by intussusception as well [9]. Reports of metastasis of Ewing sarcoma to the small bowel are even fewer and far between. Capitini reported a case of left femur Ewing sarcoma metastasizing to the brain and small intestine after allogenic stem cell transplantation [13].

## Case presentation

A previously well 44-year-old female presented with acute lower limb numbness and weakness of 1-day duration. Prior to this, she suffered from back pain for 2 weeks that gradually progressed to pain in both hips as well. There were no complaints of urinary or bowel incontinence. She did not experience any abdominal pain, distension, vomiting or constipation.

On examination, the patient had decreased sensation and power in the bilateral lower limbs and up-going plantar reflexes. Abdominal examination revealed no mass or distension. Bowel sounds were active. Anal tone was intact on digital rectal examination.

**Fig. 1:**
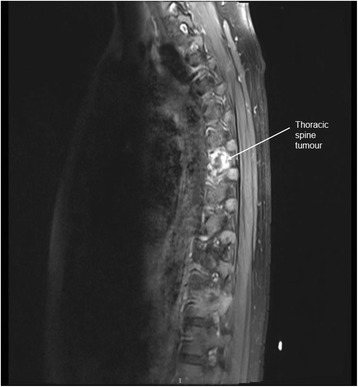
MRI spine showing abnormal enhancement of the thoracic vertebra with spinal cord compression

**Fig. 2:**
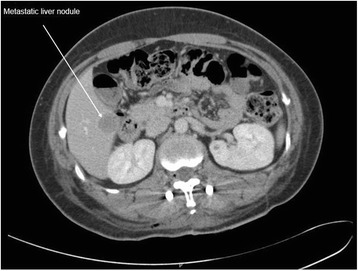
Liver metastatic nodule on initial CT

**Fig. 3:**
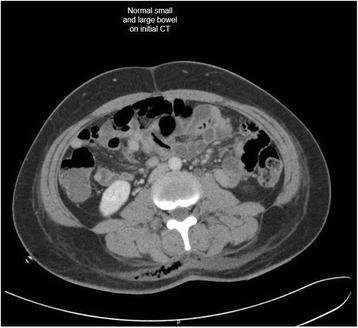
Normal bowel on initial CT

Magnetic resonance imaging of her thoracic and lumbar spine with 10 ml intravenous Magnevist showed abnormal marrow replacement affecting the 11th thoracic vertebral body associated with enhancement causing compression of the spinal cord (Fig. 1). The initial impression was spine metastases from unknown primary causing spinal cord compression. The patient underwent excision of thoracic spine tumour, decompression laminectomy and fixation the following day. A computed tomography (CT) scan of her thorax and abdomen with 80 ml intravenous Omnipaque 350 was performed 2 days after surgery to look for a primary malignancy. A 2-cm liver lesion (Fig. 2) was detected along with an indeterminate 0.3-cm left pulmonary nodule and a single enlarged 1.3-cm para-aortic lymph node. The small and large bowels were normal (Fig. 3). Immunohistology of the vertebral tumour revealed epidural Ewing sarcoma. Molecular testing detected presence of t(11;22)(q24;q12) translocation further confirming the diagnosis of Ewing sarcoma.

**Fig. 4:**
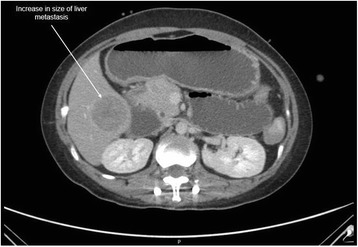
Increase in size of metastatic liver nodule on subsequent CT

**Fig. 5:**
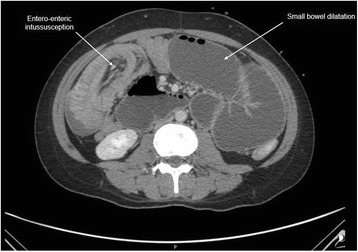
New development of entero-enteric intussusception and small bowel dilatation on subsequent CT

**Fig. 6:**
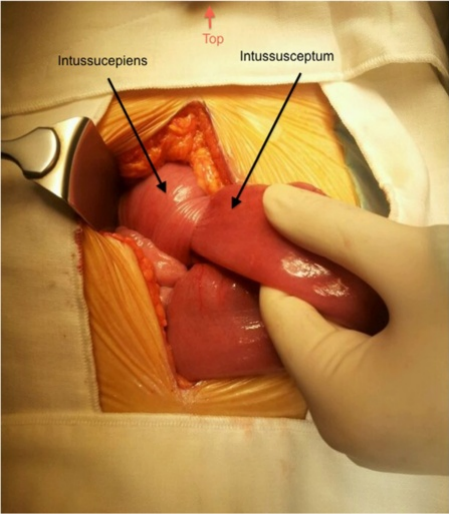
Intra-operative findings of jejunal-jejunal intussusception

**Fig. 7:**
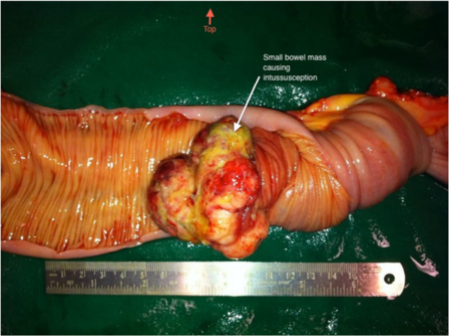
Intraluminal mass forming pathologic lead point

**Fig. 8:**
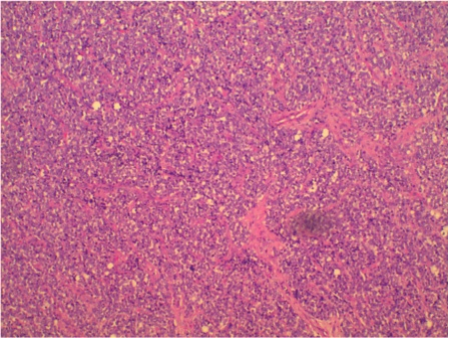
Characteristic sheets of small blue round cells from primary vertebral Ewing sarcoma

**Fig. 9:**
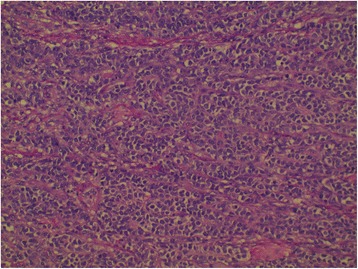
Histomorphologically similar round cells from small bowel metastatic tumour

**Fig. 10:**
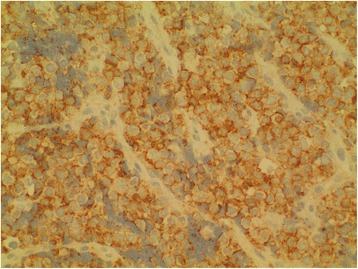
Positive cell membrane reactivity for CD99 from primary vertebral Ewing sarcoma

**Fig. 11:**
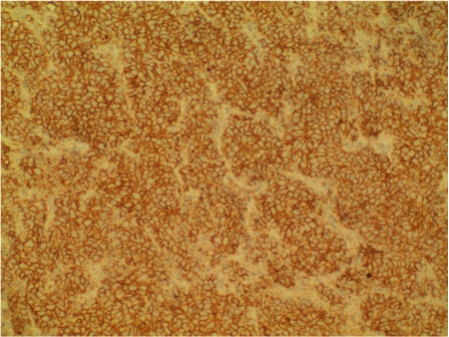
Positive CD99 staining from small bowel metastatic tumour

**Fig. 12:**
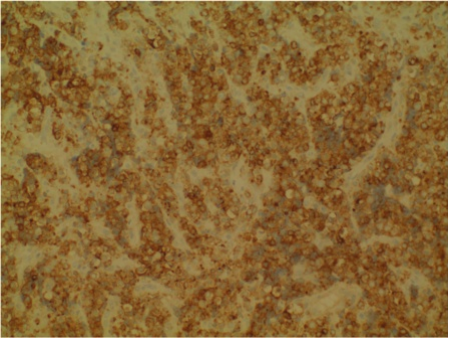
Positive staining for neuroendocrine marker synaptophysin in primary vertebral Ewing sarcoma

**Fig. 13:**
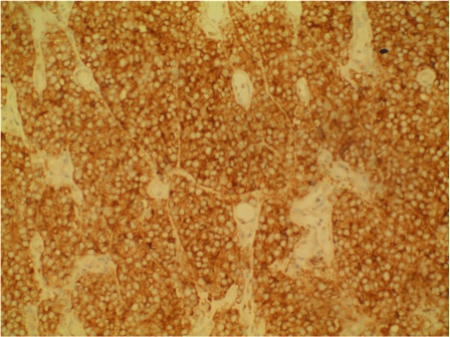
Positivity of neural marker synaptophysin in small bowel metastatic tumour

The patient was planned for chemotherapy but, 3 weeks after her spine surgery, she developed symptoms of intestinal obstruction with persistent vomiting, intermittent upper abdominal pain and distension. A repeat CT scan showed multiple new lytic lesions in the vertebrae, right iliac bone and right femur, increase in size and number of liver nodules (Fig. 4) and para-aortic lymphadenopathy. It also showed new findings of small bowel dilatation secondary to an entero-enteric intussusception (Fig. 5). An emergent laparotomy was performed and jejunal-jejunal intussusception was found (Fig. 6) with a 5-cm tumour forming the lead point (Fig. 7). There were no other lesions in the small bowel and colon. The affected segment of the small bowel was resected with primary anastomosis. Histopathological examination of the resected small bowel tumour confirmed metastatic Ewing sarcoma, morphologically similar to the spinal tumour (Figs. 8 and 9).The entire panel of immunochemical stains including CD99, synaptophysin, chromogranin, MNF-116, AE1/AE3, epithelial membrane antigen (EMA), CD34, HMB-45,desmin and S100 showed similar results in both the vertebral as well as the small bowel tumour (Figs. 10, 11, 12 and 13).

The patient made an uneventful recovery from surgery and was started on palliative chemotherapy. She eventually succumbed to progressive metastatic disease 16 months after her initial diagnosis.

### Discussion

The Ewing sarcoma family of tumors (ESFT) consists of Ewing sarcoma, peripheral primitive neuroectodermal tumor (PNET), extraosseous Ewing sarcoma (EES), and Askin’s tumor (Ewing sarcoma of the chest wall). ESFT tumours are of neural crest derivation that differentiate along a neuroendocrine lineage and are described as small round cell tumours. All ESFT tumours are characterized by a balanced chromosomal translocation between the 5ʹ half of the *EWS* gene (22q12) and the 3ʹ half of members of the *ETS* family of transcription factors, leading to the understanding that ESFT represents a single neoplastic entity [5].

Ewing sarcoma predominantly affects children and young adults with a peak incidence between 10 and 20 years of age. About 30 % occur in adults over the age of 20 and fewer than 5 % occur in adults over the age of 40 [6]. Ewing sarcoma most often arises in the mid-shaft or diaphysis of the long bones of the extremities with the spine making up only 8 % of primary sites of Ewing sarcoma [2]. Our patient, being a 44-year-old female adult, did not fit the typical profile of an Ewing sarcoma patient and was initially treated as spinal metastases from a yet undetermined primary. It was only on histopathological examination of the spinal tumour that the diagnosis of Ewing sarcoma was reached.

The presence of metastasis is the single most important factor in determining survival in ESFT patients. Patients with metastatic disease at diagnosis have a dismal 5-year survival rate varying from 0–25 %, compared with 40–79 % for those with localized disease [5, 7]. Chemotherapy is essential in the treatment of Ewing sarcoma because although approximately 80 % of patients present with clinically localized disease, subclinical metastatic disease is presumed to be present in almost all patients due to a 80–90 % relapse rate noted in patients who underwent local therapy alone [3]. It is possible that our patient had occult metastases that continued to rapidly progress after excision of the primary tumour before commencement of systemic chemotherapy. Metastases are mostly found in the lungs (50 %), bone (25 %) and bone marrow (20 %) [4]. Only eight cases of primary Ewing sarcoma of the small bowel have been reported [8–10, 14, 15], and metastasis of Ewing sarcoma to the small bowel is even rarer with Capitini reporting a case of a 26-year-old male with metastasis of left femur Ewing sarcoma to the small bowel and brain following allogenic stem cell transplantation.

**Fig. 14:**
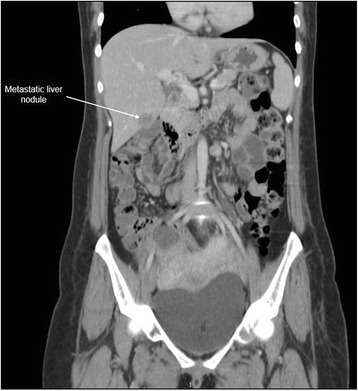
Initial coronal CT showing normal appearance of bowel and liver metastasis

**Fig. 15:**
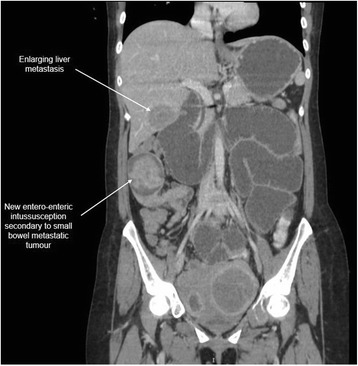
Subsequent coronal CT showing new entero-enteric intussusception and enlargement of liver metastasis

Intussusception is the invagination of a bowel loop with its mesenteric fold (intussusceptum) into the lumen of a contiguous portion of the bowel (intussuscipiens) as a result of peristalsis. Intussusception is rare in adults, accounting for 1–5 % of all cases of intestinal obstruction and 5 % of all intussusceptions [11]. Most cases of intussusception in adults are due to a pathologic lead point within the bowel and are malignant in over 50 % of cases [12], thereby necessitating surgery and resection of the affected bowel segment. In the small intestine, an intussusception can be secondary to the presence of intra- or extra-luminal lesions which include inflammatory lesions, Meckel’s diverticulum, postoperative adhesions, lipoma, adenomatous polyps, lymphoma and metastases or iatrogenic (the presence of an intestinal tube, in patients with a gastrojejunostomy, etc.). Malignancy (adenocarcinoma) accounts for up to 30 % of cases of intussusception occurring in the small bowel [11]. In 2003, Boehm [9] described a case of ileoileal intussusception in an 18-year-old male who presented with a protracted course of abdominal pain and vomiting for several weeks. The intussusception was eventually discovered on laparotomy and histopathology of the resected bowel revealed primary Ewing sarcoma. Our patient suffered from intussusception as well, although the cause was due to a large jejunal intraluminal metastasis from her primary spinal ES. No bowel mass was evident on her initial CT scan (Figs. 3 and 14), but her subsequent CT performed after development of intestinal obstructive symptoms showed not only new development of small bowel intussusception but also progression of metastatic disease (Figs. 4, 5 and 15). The resected jejunal metastatic tumour, possibly a result of haematogenous spread, displayed the same histomorphological and immunohistochemical characteristics as the vertebral primary tumour (Figs. 8, 9, 10, 11, 12 and 13).

## Conclusions

Ewing’s sarcoma is a highly aggressive small round cell tumour that rarely arises in adults. The presence of metastases, overt or subclinical, is thought to be present in almost all patients at diagnosis and, as evidenced by our patient, can progress rapidly to cause further complications and confer a poorer survival. The possibility of metastasis, no matter how rare or unlikely the site is, should be considered and actively investigated to expedite treatment of the primary disease.

## Consent

Written informed consent was obtained from the patient for the publication of this report and any accompanying images. A copy of the written consent is available for re-view by the Editor-in-Chief of this journal.
